# Inadequate Vitamin C Status in Prediabetes and Type 2 Diabetes Mellitus: Associations with Glycaemic Control, Obesity, and Smoking

**DOI:** 10.3390/nu9090997

**Published:** 2017-09-09

**Authors:** Renée Wilson, Jinny Willis, Richard Gearry, Paula Skidmore, Elizabeth Fleming, Chris Frampton, Anitra Carr

**Affiliations:** 1 Department of Medicine, University of Otago, Christchurch 8011, New Zealand; renee.wilson@postgrad.otago.ac.nz (R.W.); richard.gearry@otago.ac.nz (R.G.); chris.frampton@otago.ac.nz (C.F.); 2 Lipid and Diabetes Research Group, Canterbury District Health Board, Christchurch 8011, New Zealand; jinny.willis@cdhb.health.nz; 3 Department of Human Nutrition, University of Otago, Dunedin 9016, New Zealand; paula.skidmore@otago.ac.nz (P.S.); liz.fleming@otago.ac.nz (E.F.); 4 Department of Pathology, University of Otago, Christchurch 8011, New Zealand

**Keywords:** vitamin C, glycaemic control, metabolic health, prediabetes, type 2 diabetes mellitus

## Abstract

Vitamin C (ascorbate) is an essential micronutrient in humans, being required for a number of important biological functions via acting as an enzymatic cofactor and reducing agent. There is some evidence to suggest that people with type 2 diabetes mellitus (T2DM) have lower plasma vitamin C concentrations compared to those with normal glucose tolerance (NGT). The aim of this study was to investigate plasma vitamin C concentrations across the glycaemic spectrum and to explore correlations with indices of metabolic health. This is a cross-sectional observational pilot study in adults across the glycaemic spectrum from NGT to T2DM. Demographic and anthropometric data along with information on physical activity were collected and participants were asked to complete a four-day weighed food diary. Venous blood samples were collected and glycaemic indices, plasma vitamin C concentrations, hormone tests, lipid profiles, and high-sensitivity C-reactive protein (hs-CRP) were analysed. A total of 89 participants completed the study, including individuals with NGT (*n* = 35), prediabetes (*n* = 25), and T2DM managed by diet alone or on a regimen of Metformin only (*n* = 29). Plasma vitamin C concentrations were significantly lower in individuals with T2DM compared to those with NGT (41.2 µmol/L versus 57.4 µmol/L, *p* < 0.05) and a higher proportion of vitamin C deficiency (i.e. <11.0 µmol/L) was observed in both the prediabetes and T2DM groups. The results showed fasting glucose (*p* = 0.001), BMI (*p* = 0.001), smoking history (*p* = 0.003), and dietary vitamin C intake (*p* = 0.032) to be significant independent predictors of plasma vitamin C concentrations. In conclusion, these results suggest that adults with a history of smoking, prediabetes or T2DM, and/or obesity, have greater vitamin C requirements. Future research is required to investigate whether eating more vitamin C rich foods and/or taking vitamin C supplements may reduce the risk of progression to, and/or complications associated with, T2DM.

## 1. Introduction

Type 2 diabetes mellitus (T2DM) is a complex disorder influenced by both genetic and environmental factors. It is characterized by chronic hyperglycemia, altered insulin secretion, and insulin resistance [1]. As in many Western countries, T2DM is associated with increased morbidity and mortality due to microvascular (e.g. retinopathy, nephropathy, and neuropathy) and macrovascular complications (e.g. myocardial infarction, peripheral vascular disease, and stroke) [1]. Diabetes is one of the largest global health emergencies with 415 million people between the ages of 20 and 70 worldwide estimated as having diabetes in 2015 and the prevalence is increasing [2]. T2DM accounts for at least 90% of all cases of diabetes [2]. In 2016, approximately 5% of New Zealanders were living with diabetes compared to an estimated 6.5% of people in the UK [3,4].

Research suggests that chronic low grade inflammation and oxidative stress plays a pivotal role in the development of insulin resistance and T2DM, as well as the related complications [5]. Vitamin C is an essential micronutrient with potent antioxidant properties [6]. Vitamin C can protect important biomolecules from oxidation through participating in oxidation-reduction reactions whereby it is readily oxidized to dehydroascorbic acid, which in turn is rapidly reduced back to ascorbate [7]. Vitamin C is naturally present in fruit and vegetables, is often added as a preservative to foods/beverages, and is also used as a dietary supplement [6]. As a result of being water-soluble, it has a relatively short half-life in the body due to rapid renal clearance and a regular and adequate intake is required to prevent deficiency.

Previous research suggests that people with T2DM have lower plasma vitamin C concentrations than those with normal glucose control [8,9,10]. There are several proposed mechanisms including: (1) increased ascorbate excretion in those with microalbuminuria, (2) blood glucose may compete with vitamin C for uptake into cells due to its structural similarity to the oxidised form (dehydroascorbic acid), and (3) increased oxidative stress may deplete antioxidant stores [8]. Recent research has indicated that the glucose-dependent inhibition of dehydroascorbic acid uptake into erythrocytes may contribute to enhanced erythrocyte fragility and could potentially contribute to complications such as diabetic microvascular angiopathy [11].

As dietary vitamin C contributes to plasma vitamin C concentrations, potential differences in the intake between those with normal glucose control and T2DM must also be considered. A prospective study of 48,850 men revealed that while the baseline consumption of fruit and vegetables was similar, men who developed T2DM increased their consumption of fruit and vegetables by 1.6 serves/week compared to an increase of 0.7 serves/week in those who remained diabetes free [12]. Therefore, it seems that people with T2DM are altering their diet in an attempt to manage their blood sugar. Indeed, clinical advice to those newly-diagnosed with T2DM focuses on improving the diet. However, the dietary changes appear to be small and, furthermore, those with T2DM appear to have a similar intake of fruit and vegetables to those without T2DM [12].

The lower plasma vitamin C concentrations reported in people with T2DM has led to a growing interest in the role that vitamin C may afford against the development of T2DM and associated complications. A prospective survey of the Dutch and Finnish cohorts within the Seven Countries Study revealed an inverse association between dietary vitamin C intake and glucose intolerance, suggesting that antioxidants such as vitamin C may play a protective role against the development of impaired glucose tolerance and T2DM [13]. Further, the European Prospective Investigation of Cancer (EPIC)-Norfolk Study of some 21,000 individuals ascertained 735 cases of T2DM after a 12 year follow-up, and demonstrated a strong inverse association between plasma vitamin C concentration and T2DM risk [14].

However, studies investigating plasma vitamin C and glycaemic control have often failed to account for factors such as smoking status and dietary vitamin C intake, which are known to impact plasma vitamin C concentrations. When dietary intake is taken into account there are conflicting results, with one study showing a low plasma vitamin C concentration in people with diabetes consuming a similar amount of dietary vitamin C to those without diabetes [15], compared to another study that reported no differences in serum vitamin C concentrations in people grouped by T2DM status after adjustment for dietary vitamin C intake [16]. Therefore, the objective of this study was to determine the association between plasma vitamin C status and glycaemic control accounting for vitamin C intake in adults.

## 2. Materials and Methods

### 2.1. Study Participants

This study was approved by the New Zealand Central Health and Disability Ethics Committee (consent no. 14/CEN/34). Written informed consent was obtained from all participants. Individuals aged ≥ 18 years meeting the inclusion criteria detailed below were recruited from General Practice, Prediabetes and Diabetes Services, Retinal Screening Services, Pharmacies, and from local advertisements. Fasting glucose cut-off values for normal glucose tolerance (NGT), prediabetes, and T2DM were based on the American Diabetes Association (ADA) criteria [1]. Those taking Metformin were also included in the T2DM group. A total of 101 individuals underwent a screening questionnaire to ascertain the eligibility for the study. Ninety participants were enrolled and 89 participants completed the study. One participant was excluded due to incomplete sample collection.

### 2.2. Study Design

This was a cross-sectional observational pilot study that was part of a wider study on the gut microbiota and glycaemic control. At their study appointment, participants completed demographic and physical activity questionnaires. Anthropometric data were collected including the body mass index (BMI), waist and hip circumference, and bioelectrical impedance. The completed four-day weighed food diary was reviewed and additional information was added if necessary. A venous blood sample was also collected after an overnight fast and the blood pressure was measured.

#### 2.2.1. Inclusion Criteria

Individuals aged ≥18 years with: NGT (fasting glucose ≤5.5 mmol/L) (*n* = 35), prediabetes (fasting glucose ≥5.6 mmol/L) (*n* = 25), T2DM taking no diabetes medication (fasting glucose ≥7.0 mmol/L) or on a regimen of Metformin only (*n* = 29).

#### 2.2.2. Exclusion Criteria

Individuals unable to give informed consent, those who had taken antibiotics in the last month, those with a medical history of significant gastrointestinal disease e.g. inflammatory bowel disease, those who had undergone a previous bowel resection, and individuals taking diabetes medication other than Metformin.

### 2.3. Demographic Information

Participants recorded their date of birth, sex, ethnicity, qualification, and smoking status. They also recorded information on current medication and supplement use.

### 2.4. Anthropometric Measures

Weight (kg). Participants were asked to remove their footwear and heavy outer clothing such as jackets and were weighed to the nearest 0.1 kg on calibrated Tanita scales (Model BWB-800A, Tanita Corporation, Tokyo, Japan).

Height (m). Measured once to the nearest mm using calibrated height measures.

BMI (kg/m^2^). Widely accepted as an appropriate population-level indicator of excess body fat [17]. BMI is calculated by weight in kilograms divided by height in metres squared.

Waist circumference and the waist-to-hip ratio are alternative anthropometric measures that also indicate whether excess body fat is centrally or peripherally located.

Waist circumference (cm). The World Health Organisation (WHO) STEPwise Approach to Surveillance (STEPS) protocol for measuring the waist circumference was used. The measurement was made at the approximate midpoint between the lower margin of the last palpable rib and the top of the iliac crest [18]. The tightness of the tape was controlled by using a Gulick II Measuring tape (Model 67020, Country Technology Inc, Gays Mills, Wisconsin, WI, USA). Two to three measures were recorded and if the difference between the measurements exceeded 1.5 cm, a third measure was taken. The measures for each participant were averaged.

Hip circumference (cm). Measured to the nearest mm around the widest portion of the buttocks with the tape parallel to the floor using a Gulick II Measuring tape, as described above.

Waist-to-hip ratio. Calculated by dividing the waist circumference by the hip measurement.

Fat mass (%). Measured using the BIA 450 Bioimpedance Analyser (Biodynamics Corporation, Seattle, Washington, DC, USA). Patient assessments were conducted using a connection between the individual’s wrist and ankle and the analyser using standard ECG sensor pad electrodes (CONMED Corporation, Utica, New York, NY, USA).

Blood Pressure. Measured using an automated blood pressure monitor (Bp TRU, BTM-300, Omron Healthcare Co., Ltd, Muko, Kyoto, Japan). The measurement was repeated if the results were outside the normal range. If there was an obvious outlier, this result was removed and the other results were averaged.

### 2.5. Blood Parameters

Venous blood samples were collected after a 12–hour fast.

Glycated haemoglobin (HbA1c). Determined in EDTA blood by standard methods (Bio-rad Variant HPLC, Bio-Rad, Hercules, California, CA, USA) at an International Accreditation New Zealand (IANZ) laboratory.

Glucose. Fasting glucose was measured in blood collected in fluoride oxalate venoject tubes by standard methods (Glucose Hexokinase Enzymatic Assay, Abbott c series analyser, Abbott Park, Illinois, USA) at an IANZ laboratory.

Lipid parameters. Total cholesterol (TC), HDL-cholesterol (HDL), LDL-cholesterol (LDL), and triglycerides (TG) were determined in lithium heparin blood by standard methods (Abbott c series analyser, Abbott Park, Illinois, IL, USA) at an IANZ laboratory.

High-sensitivity C-reactive protein (hs-CRP). The inflammatory marker hs-CRP was measured using end-point nephelometry at an IANZ laboratory.

Plasma vitamin C and hormones. EDTA blood was collected and centrifuged for 15 minutes at 1500 g at 4 ˚C. The plasma was stored −80 ˚C prior to analysis.

#### 2.5.1. Plasma Vitamin C

Stored plasma was rapidly thawed, and acidified with perchloric acid and a metal chelator (DTPA) to precipitate the protein and stabilise the ascorbate [19]. Following centrifugation, the supernatant was treated with a reducing agent (TCEP) to recover any ascorbate that had become oxidised during the processing and storage of the samples [20]. The vitamin C concentration of the processed samples was determined using high performance liquid chromatography (HPLC) with electrochemical detection in the Department of Pathology, University of Otago Christchurch, as described previously [19].

#### 2.5.2. Plasma Ghrelin, Leptin, and Adiponectin

Plasma hormones were determined by the Christchurch Heart Institute, Department of Medicine, University of Otago, Christchurch.

Plasma ghrelin was measured by an in-house radioimmunoassay (RIA) following extraction from plasma using Sep Pak C_18_ cartridges, as described previously [21]. The assay recognises the total circulating ghrelin (i.e. both octanoyl and non-octanoyl forms). The cross reactivities of other peptides in the assay, including vasointestinal peptide, prolactin, galanin, growth hormone releasing hormone, neuropeptide Y, brain natriuretic peptide, atrial natriuretic peptide, endothelin-1, and angiotensin II were all less than 0.03%. The RIA had a mean detection limit of 10.8 ± 0.8 pmol/L and mean ED_50_ of 136.2 ± 10.0 pmol/L over 23 consecutive assays.

Plasma leptin and adiponectin were measured using commercial enzyme-linked immunosorbent assays (ELISA) from BioVendor (Brno, Czech Republic), Research and Diagnostic products (RD191001100 Human Leptin ELISA and RD191023100 Human Adiponectin ELISA) according to the manufacturer’s instructions.

#### 2.5.3. Plasma Insulin

Plasma insulin was measured using the Roche Cobas e411 method in an IANZ laboratory. After storage at -80 ˚C, thawed plasma was pre-treated using 25% polyethylene glycol to precipitate any unwanted antibodies.

### 2.6. Dietary Intake of Vitamin C, Macronutrients, and Fibre

Participants completed a four day (non-consecutive) weighed food diary (including one weekend day) prior to their study visit. Participants received training using the Salter digital scales and on how to record the data, either at home or in the clinic, prior to the diary being completed. Once completed, the diary was also reviewed at their second study visit to add any missing information if necessary. The food diaries were entered into the nutrient analysis programme Kai-culator (version 1.08d, Department of Human Nutrition, University of Otago, Dunedin, New Zealand). Kai-culator uses the 2014 version of the New Zealand food composition database “NZ FOODfiles”. The methodology for entering the diaries was developed by a dietitian and data entry was undertaken by an experienced dietitian and an experienced nutritionist who cross-checked each other’s data and were overseen by an experienced nutritionist and dietitian. A further 16% of the diaries were checked again for accuracy. The Acceptable Macronutrient Distribution Ranges (AMDR) are the recommendations for the balance of protein, fat, and carbohydrate in the diet with respect to the relative contribution to dietary energy [22]. Total daily vitamin C, energy, and fibre were calculated, along with the percent energy values for fat, carbohydrate, and protein. Participants were asked to record the name of dietary supplements taken within the last month, the amount per dose, the frequency, when they started taking the supplement, and when their last dose was.

### 2.7. Physical Activity

Participants completed the self-administered short form version of the International Physical Activity Questionnaire (IPAQ). The questionnaire asks about physical activity over the previous seven days.

### 2.8. Statistical Analyses

Standard descriptive statistics including means, standard deviations, frequencies, and percentages as appropriate were used to summarise the demographic, anthropometric, and laboratory results across participants grouped by fasting glucose and T2DM treatment. Four of the laboratory measures (hs-CRP, Ghrelin, Leptin, and Adiponectin) showed a strong positive skew and were therefore log_e_ transformed prior to analyses. These variables are described using geometric means and 95% confidence intervals. Associations between the clinical characteristics of the cohorts grouped by fasting glucose (including those treated with Metformin) were tested using one way analysis of variance (ANOVA) and chi-squared tests as appropriate. Where significant associations were identified, these were further explored with pair-wise comparisons amongst the fasting glucose groups. The univariate associations between plasma vitamin C and demographic, anthropometric, and laboratory measures were tested using Pearson’s Correlations coefficients and one way ANOVA. Significant predictors identified from these univariate analyses were then combined in a multiple regression analysis to identify significant independent associations with plasma vitamin C. The two-tailed *p*-value < 0.05 was taken to indicate statistical significance. All statistical analyses were undertaken using SPSS (version 24.0, IBM Corp., Armonk, New York, NY, USA).

## 3. Results

### 3.1. Participant Characteristics

The NGT group was slightly younger than the prediabetes and T2DM groups and there were more females in the NGT group and less in the T2DM group. The majority of participants were European and there were a mix of qualifications, as would be expected given the age of the participants (Table 1). There were no significant differences in physical activity between the study groups although those in the NGT and prediabetes groups had reported slightly higher levels of activity than those with T2DM.

The mean BMI for the NGT and prediabetes groups reflects the international BMI cut-off points for overweight (25.00–29.99 kg/m^2^) and the T2DM group were obese (≥30.00 kg/m^2^). The waist circumference and waist-to-hip ratio increased across the groups from NGT to T2DM along with fat mass (%), as would be expected given that obesity is a risk factor for T2DM.

### 3.2. Metabolic and Inflammatory Plasma Biomarkers

Glycaemic measures (fasting glucose and HbA1c), used as the basis for defining prediabetes and T2DM, increased from NGT to T2DM as expected and differed significantly between the study groups (*p* < 0.05, Table 2). Although fasting glucose was used as the basis for classifying participants in the analysis, the mean HbA1c of 35 mmol/mol for the NGT group and 40 mmol/mol for the prediabetes group were consistent with the New Zealand guidelines for the classification of diabetes based on HbA1c [23]. The mean HbA1c of 47 mmol/mol for the T2DM group is lower than the current threshold for the diagnosis of diabetes in New Zealand (50 mmol/mol) using this measure because some of the individuals in this category were treated with the biguanide oral hypoglycaemic drug, Metformin. Fasting and postprandial glucose were likely reduced in these treated individuals. The mean HbA1c for all participants was 41 mmol/mol (Table 2). While hs-CRP was inversely associated with glycaemic control, this was not significant.

The average fasting insulin concentrations were consistent with the glycaemic measures and were significantly higher in the T2DM group compared to the NGT group. The increasing BMI across the groups was associated with the increase in leptin concentrations, and the reduction in ghrelin concentrations.

Total, HDL, and LDL cholesterol decreased from the NGT to the T2DM group, which may reflect the use of lipid lowering medications which are routinely used in individuals with T2DM.

There was a slight increase in TG across the groups, with the average for each group remaining below the recommended cut-off in New Zealand (<1.7 mmol/L). There were no significant differences in the total cholesterol/HDL ratio between groups, and each group was below the recommended cut-off in New Zealand of 4.5.

### 3.3. Dietary Intake of Vitamin C, Macronutrients, and Fibre

There were no significant differences in macronutrient intake and dietary vitamin C intake across the groups (Table 3). The AMDR range for protein is 15–25% of total energy, total fat 20−35% of total energy, and carbohydrate 45–65% of total energy [22]. All study groups had slightly higher average total fat intakes and slightly lower CHO intakes than recommended, but the average protein intake for all groups fell within the recommended range.

The adequate intake (AI) for dietary fibre in New Zealand and Australia is set at the median for dietary fibre intake recorded in the 1995 National Nutrition Survey of Australia (ABS 1998) and the 1997 National Nutrition Survey of New Zealand (MOH 1999) [22]. The AI is 25 g for women and 30 g for men. Although fibre intake is not reported by sex in Table 1, the average daily fibre intake for each group of 24 g, 25 g, and 27 g for the NGT, prediabetes, and T2DM groups, respectively, was similar to recommendations.

Six participants reported taking a high dose vitamin C supplement (≥500 mg vitamin C). The plasma vitamin C concentration of five of these participants ranged from 36–59 µmol/L, which reflects inadequate to adequate plasma vitamin C concentrations and suggests that they didn’t take the supplement close to their study appointment. The other participant had a plasma vitamin C concentration of 74 µmol/L, which is a saturating concentration, but they also had an average dietary vitamin C intake of 194 mg/day and so this high plasma concentration could be explained by their dietary intake as 200 mg/day will saturate plasma [19].

### 3.4. Plasma Vitamin C Status and Dietary Vitamin C Intakes

**Figure 1:**
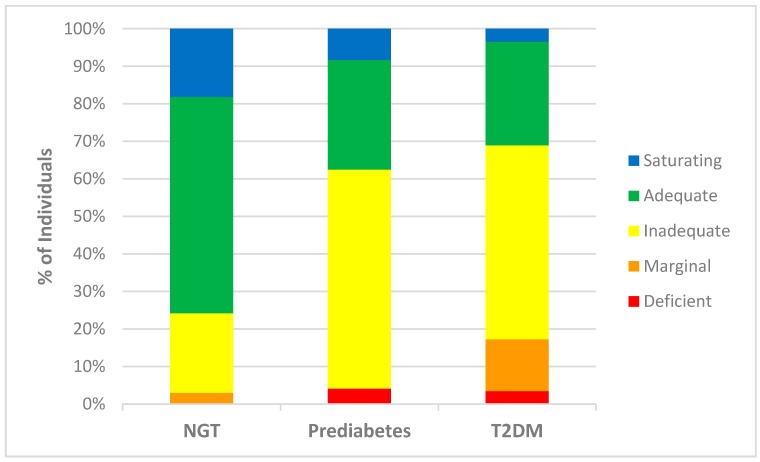
Plasma vitamin C status of individuals within study groups. Percentage of individuals from each study group [normal glucose tolerance (NGT), prediabetes, and type 2 diabetes mellitus (T2DM), including those taking no diabetes medication (fasting glucose ≥ 7.0 mmol/L or on a regimen of Metformin only (T2DM)], classified as having saturating (>70 µmol/L), adequate (51.0–69.9 µmol/L), inadequate (24.0–50.9 µmol/L), marginal (11.0–23.9 µmol/L), and deficient (<11.0 µmol/L) plasma vitamin C concentrations [24].

A significant decrease in the mean plasma vitamin C concentration was observed between the NGT (57.4 µmol/L) and the prediabetes group (48.2 µmol/L) (*p* = 0.035) and the T2DM (41.2 µmol/L) group (*p* < 0.001) (Table 2). Furthermore, there was a much higher proportion of individuals with prediabetes and T2DM with deficient (4% and 3% respectively), marginal (14% in T2DM group), and inadequate (58% in prediabetes and 52% in T2DM group) plasma vitamin C concentrations, compared with the NGT group (3% marginal and 21% inadequate) (Figure 1).

**Figure 2:**
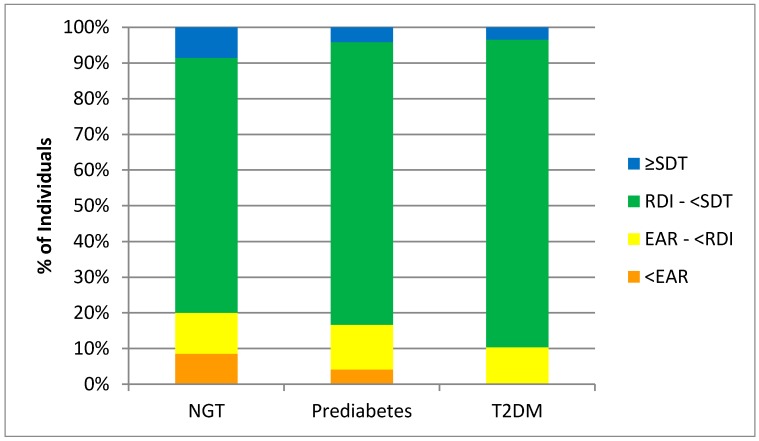
Individuals meeting New Zealand dietary intake recommendations for vitamin C. Percentage of individuals from each study group [normal glucose tolerance (NGT), prediabetes, and type 2 diabetes mellitus (T2DM), including those taking no diabetes medication (fasting glucose ≥7.0 mmol/L or on a regimen of Metformin only (T2DM)], meeting the estimated average requirement (EAR) (30 mg/day), recommended dietary intake (RDI) (45 mg/day), and suggested dietary target (SDT) to reduce chronic disease risk (220 mg/day for men and 190 mg/day for women) for dietary vitamin C intake using the nutrient reference values for Australia and New Zealand [22].

Although plasma vitamin C decreased from NGT to T2DM, there were no significant differences in dietary vitamin C concentrations between study groups determined from the four day weighed food diaries (Table 3). The majority of participants met the New Zealand recommended dietary intake (RDI) of 45 mg/day (Figure 2). Furthermore, there were no participants in the T2DM group that had intakes below the New Zealand estimated average requirement (EAR) (30 mg/day). At the group level, it appears that most participants had an adequate fruit and vegetable intake to meet the recommended vitamin C intakes. However, few participants were reaching the New Zealand Ministry of Health’s suggested dietary targets (SDT) to reduce chronic disease risk, i.e. 220 mg/day for men and 190 mg/d for women (Figure 2).

### 3.5. Plasma Vitamin C Correlations

There were no significant associations between age, gender, ethnicity, education level, and plasma vitamin C concentrations. There was a significant association between smoking history and plasma vitamin C concentration (*p* = 0.035), with current (mean 30.9 µmol/L) and ex-smokers (mean 47.3 µmol/L) having lower concentrations than non-smokers (mean 52.6 µmol/L). There was a significant linear association between vitamin C intake and plasma vitamin C concentration (*r* = 0.353, *p* = 0.001).

The three anthropometric measures (BMI, fat mass, and waist-to-hip ratio) were all significantly negatively associated with plasma vitamin C (*p* < 0.05) when conducting univariate analyses. When these three variables were included in a multiple regression, only BMI was independently negatively associated with plasma vitamin C (*p* < 0.001). Laboratory measurements (HbA1c, fasting glucose, TG, total chol/HDL chol, insulin, and hs-CRP) were negatively associated with plasma vitamin C (*p* < 0.05) and HDL chol and ghrelin were positively associated with plasma vitamin C (*p* < 0.05) in the univariate anlaysis (Table 4). When these variables were included in a multiple regression, only hs-CRP and fasting glucose were independently negatively associated with plasma vitamin C (*p* < 0.05).

A final multiple regression showed fasting glucose (*p* = 0.001), BMI (*p* = 0.001) and smoking history (*p* = 0.003) to be significant independent predictors of plasma vitamin C. Fasting glucose and BMI were negatively associated with plasma vitamin C, and current and ex-smokers had reduced plasma vitamin C concentrations compared to non-smokers. There was a strong positive association between hs-CRP concentrations and BMI (*r* = 0.618, *p* < 0.001). Accordingly, hs-CRP does not feature as an independent predictor of plasma vitamin C. Including dietary vitamin C intake in the above model (Table 5) showed that this was a significant independent predictor (*p* = 0.032) of plasma vitamin C concentrations, and BMI, fasting glucose, and smoking history remained as significant independent predictors (*R*^2^ = 0.43).

## 4. Discussion

### 4.1. Predictors of Plasma Vitamin C

This study showed fasting glucose, BMI, smoking history, and dietary vitamin C intake to be significant independent predictors of plasma vitamin C concentrations. The inverse association between fasting glucose and plasma vitamin C concentration shown in this study is in agreement with earlier studies [8,9,10]. In addition, the mean plasma vitamin C concentration was significantly lower in the prediabetes group (compared to the NGT group, suggesting that a reduction in plasma vitamin C concentration occurs in parallel with the decline in glucose tolerance during the progression to T2DM. It has been proposed that the uptake of dehydroascorbic acid, the oxidized form of vitamin C, by the glucose transporters (GLUTs), could be competitively inhibited by elevated blood glucose levels [25]. This could contribute to complications such as diabetic microvascular angiopathy due to erythrocyte fragility, as erythrocytes lack the sodium-dependent vitamin C transporters (SVCTs) and are dependent on the GLUTs for the uptake of vitamin C [11]. Our study also found plasma vitamin C concentration to be inversely related to BMI, which concurs with previous research [26]. Individuals with a higher weight are prone to vitamin C inadequacy and are known to require higher intakes of vitamin C in order to reach adequate plasma concentrations [27,28].

Oxidative stress is defined as a significant imbalance between the production of reactive oxygen species (ROS) and antioxidant defenses, and leads to alterations in signalling pathways and to potential tissue damage [29]. ROS activate nuclear factor кB (NFкB), a pro-inflammatory transcription factor, triggering a signalling cascade that leads to the continued synthesis of oxidative species and low-grade chronic inflammation [29]. High-sensitivity CRP, produced by the liver, reflects the presence of inflammation in the body. The concentration of hs-CRP increased with the deterioration of glycaemic control and increase in BMI. This result is consistent with the evidence suggesting that obesity can lead to chronic activation of the innate immune system and low-grade systemic inflammation and oxidative stress, which have been implicated in the development of insulin resistance and T2DM [5,30,31]. Hyperglycaemia, increased plasma concentrations of free fatty acids (FFAs), and hyperinsulinaemia have all been linked to an increased production of ROS [29,31]. Our data showed an inverse relationship between hs-CRP and plasma vitamin C. It is therefore hypothesized that lower plasma vitamin C in those with higher BMI, prediabetes, and T2DM reflects the depletion of the vitamin due to its antioxidant and anti-inflammatory activities.

Consistent with the role of vitamin C as an antioxidant, our data showed a significant inverse relationship between plasma vitamin C concentration and smoking status, with ex- and current-smokers having lower plasma vitamin C concentrations than non-smokers, which is consistent with previous research [32,33]. It has long been recognised that smokers and passive smokers have a lower vitamin C status than non-smokers partly due to poor dietary habits, but also due to the oxidizing properties of tobacco smoke, resulting in an increased turnover of vitamin C [34].

As expected, dietary vitamin C was found to be a predictor for plasma vitamin C concentration. However, when the dietary intake of the vitamin was corrected for by covariate analysis fasting glucose, BMI and smoking status remained as significant independent predictors of plasma vitamin C concentration. That is, the associations observed were not solely explained by differences in dietary intake. This result is at odds with one study that reported no differences in serum vitamin C concentrations in people grouped by diabetes status after adjustment for dietary vitamin C intake [16].

### 4.2. Metabolic Hormones

The average fasting insulin concentrations were consistent with the glycaemic measures and were significantly higher in the T2DM group compared to the NGT group. A higher fasting insulin concentration indicates insulin resistance, a well-known contributor to impaired glucose tolerance and T2DM. Leptin and ghrelin are two hormones that have a major influence on energy balance [35]. Leptin is a mediator of long-term regulation of energy balance, suppressing food intake and thereby inducing weight loss. Ghrelin, on the other hand, is a fast-acting hormone, seemingly playing a role in meal initiation. In obese patients, the circulating concentration of leptin is increased, whereas surprisingly, ghrelin is decreased [35]. It is now established that obese patients are leptin-resistant [35]. Indeed, in this study, the increasing BMI across the groups was associated with an increase in leptin concentrations and a reduction in ghrelin concentrations. There was an inverse relationship between insulin and leptin and plasma vitamin C, and a positive relationship between ghrelin and plasma vitamin C; however, these hormones were also associated with fasting glucose and were thus not included as independent predictors of plasma vitamin C.

### 4.3. Clinical Significance

As hyperglycemia is associated with increased oxidative stress, a role for antioxidants such as vitamin C in the prevention of T2DM and/or the reduction of complications is a reasonable proposition. Indeed, a recent meta-analysis of 15 randomized control trials (RCTs) investigating vitamin C supplementation and insulin resistance and biomarkers of glycaemic control (fasting glucose, HbA1c) found that doses of ≥200 mg/day vitamin C significantly reduced glucose concentrations in patients with T2DM, particularly if the intervention was for more than 30 days and in older individuals [36]. Furthermore, a recent 12 month RCT found that treating those with T2DM with both Metformin and vitamin C was more effective at reducing HbA1c and risk factors for diabetes-related long-term complications than treating with Metformin alone [37].

Although T2DM is not traditionally considered a risk factor for vitamin C deficiency, our research indicates that those with prediabetes or T2DM are more likely to have inadequate or deficient plasma vitamin C concentrations. This did not appear to be due to a lower dietary vitamin C intake, so dietary advice needs to emphasise the importance of consuming high vitamin C foods, aiming for an intake of at least 200 mg/day [22]. This is particularly relevant in light of the associated T2DM risk factors of higher BMI and smoking status, both of which impact vitamin C status. Further research into the possibility of a higher RDI for vitamin C for those with prediabetes and T2DM is warranted, in line with what has been recommended in some countries for smokers [38].

### 4.4. Study Strengths and Limitations

Our study used robust methodology for dietary intake, plasma vitamin C, and statistical analysis, and accounted for other factors that are known to impact plasma vitamin C concentration such as smoking status, dietary vitamin C intake, and supplement use. The participants with T2DM were clinically well defined and were either not treated with diabetes medication or treated with a single oral hypoglycaemic agent only (Metformin). Those taking Metformin were included in the overall analysis. When the Metformin treated cases were excluded, the correlation between fasting glucose and plasma vitamin C concentrations was similar in direction and magnitude (*n* = 64, *r* = −0.477, *p* < 0.001) to the entire cohort (*n* = 86, *r* = −0.411, *p* = 0.001). Further, the current norm is for Metformin treatment to be initiated at diagnosis, rather than after the failure of diet and lifestyle changes to optimize glucose control. As such, it will become increasingly difficult to recruit treatment-naïve individuals with T2DM to studies.

T2DM has been shown to increase the urinary excretion of vitamin C, leading to reduced plasma vitamin C concentrations in a rodent model [39]. Whether this also occurs in humans is unknown. In addition, the duration of T2DM was not reported. Indeed, many individuals have undiagnosed T2DM for a significant period of time prior to formal diagnosis, making it very difficult to interpret data on the duration of the disease. There are always limitations around the self-reporting of dietary data and supplement use, and the study cohort was relatively small with 89 participants. Our study had only one measure of plasma vitamin C per participant and so future research should ideally incorporate repeated samples to account for any temporal fluctuations. There was a limitation around the lack of detail with regards to vitamin C supplement use; however, only six participants reported taking high dose vitamin C supplements and the use was sporadic.

## 5. Conclusions

Our cross-sectional observational study has identified a moderate inverse relationship between plasma vitamin C and both fasting glucose and BMI in adult subjects across the glycaemic spectrum. The relationship can be explained by the depletion of vitamin C due to oxidative stress and inflammation resulting from dysglycaemia, overweight/obesity, and smoking, rather than lower dietary intakes. Further research is required to determine whether those with an increased dietary intake through fruit and vegetables and/or vitamin C supplementation have a decreased risk of progression to T2DM and/or complications associated with the metabolic syndrome and T2DM.

## Figures and Tables

**Table 1:** General characteristics of participants classified as having normal glucose tolerance (NGT) (*n* = 35), prediabetes (*n* = 25), and T2DM (*n* = 29).

Characteristics	NGT	Prediabetes	T2DM	Total
Age * (years)	55 ± 13 ^a^	63 ± 9 ^b^	61 ± 11 ^b^	59 ± 11
Sex *				
Female % (*n*)	74 (26) ^a^	52 (13) ^ab^	35 (10) ^b^	55% (49)
Male % (*n*)	26 (9)	48 (12)	66 (19)	45% (40)
**Ethnicity **				
European % (*n*)	86 (30)	88 (22)	97 (28)	90% (80)
Maori % (*n*)	9 (3)	4 (1)	3 (1)	6% (5)
Pacific Island % (*n*)	0 (0)	4 (1)	0 (0)	1% (1)
Asian % (*n*)	3 (1)	4 (1)	0 (0)	2% (2)
Other % (*n*)	3 (1)	0 (0)	0 (0)	1% (1)
**Qualification**				
No Qualification % (*n*)	96 (3)	20 (5)	25 (7)	17% (15)
Secondary School % (*n*)	20 (7)	24 (6)	32 (9)	25% (22)
Post-Secondary Certificate, Diploma or Trade Diploma % (*n*)	43 (15)	20 (5)	25 (7)	31% (27)
University % (*n*)	27 (10)	36 (9)	18 (5)	27% (24)
**Physical Activity (MET min/week)**	1723 ± 1687	2496 ± 3671	1320 ± 1490	1772 ± 2327
**Anthropometry**				
Weight * (kg)	76 ± 18 ^a^	89 ± 19 ^b^	96 ± 20 ^b^	86 ± 21
BMI * (kg/m^2^)	28 ± 6 ^a^	30 ± 7 ^ab^	33 ± 6 ^b^	30 ± 7
Fat Mass (%)	32 ± 8	33 ± 8	35 ± 7	33 ± 8
Waist Circumference * (cm)	89 ± 16 ^a^	99 ± 14 ^b^	110 ± 15 ^c^	99 ± 17
Waist-to-Hip Ratio *	0.9 ± 0.1 ^a^	0.9 ± 0.1 ^b^	1.0 ± 0.1 ^b^	0.9 ± 0.1
Blood Pressure Diastolic (mmHg)	78 ± 9	83 ± 8	79 ± 9	80 ± 9
Blood Pressure Systolic * (mmHg)	125 ± 14 ^a^	132 ± 14 ^ab^	135 ± 15 ^b^	130 ± 15
**Smoking Status**				
Current Smoker % (*n*)	7 (2)	5 (1)	3 (1)	5% (4)
Ex-smoker % (*n*)	28 (8)	439 (9)	38 (11)	35% (28)
Non-smoker % (*n*)	66 (19)	52 (11)	59 (17)	60% (47)

Values represented as mean ± SD unless stated otherwise. *All *p* values from ANOVA tests. Groups sharing a common subscript letter denotes the study groups that do not differ significantly from each other at the 0.05 level based on characteristics from Post Hoc analysis. Note: There was missing data from one participant for qualification (1 × T2DM), 12 participants for physical activity (7 × NGT and 5 × prediabetes), five participants for waist-to-hip ratio (2 × NGT and 3 × prediabetes), nine participants for blood pressure measures (4 × NGT and 5 × prediabetes), and 10 participants did not provide smoking status data (6 × NGT, 4 × prediabetes).

**Table 2:** Laboratory measures of participants classified as having normal glucose tolerance (NGT) (*n* = 35), prediabetes (*n* = 25), and T2DM (*n* = 29).

Laboratory Measures	NGT	Prediabetes	T2DM	Total
Fasting Glucose * (mmol/L)	5.0 ± 0.4 ^a^	6.2 ± 0.4 ^b^	7.2 ± 1.3 ^c^	6.0 ± 1.2
HbA1c * (mmol/mol)	35 ± 4 ^a^	40 ± 5 ^b^	47 ± 9 ^c^	41 ± 8
hs-CRP (mg/L) Mean (95% CI)	1.2 (0.9–1.6)	1.6 (1.0–2.3)	2.1 (1.4–2.8)	1.6 (1.31.9)
Total Cholesterol * (mmol/L)	5.3 ± 0.9 ^a^	5.9 ± 1.2 ^a^	4.3 ± 1.1 ^b^	5.0 ± 1.1
Cholesterol HDL * (mmol/L)	1.5 ± 0.4 ^a^	1.3 ± 0.3 ^b^	1.1 ± 0.2 ^b^	1.3 ± 0.3
Cholesterol LDL * (Calc) (mmol/L)	3.4 ± 0.8 ^a^	3.3 ± 1.0 ^a^	2.5 ± 1.0 ^b^	3.1 ± 1.0
Triglycerides * (mmol/L)	1.1 ± 0.4 ^a^	1.3 ± 0.7 ^ab^	1.4 ± 0.6 ^b^	1.3 ± 0.6
Cholesterol (total/HDL) (ratio)	3.8 ± 0.8	4.2 ± 0.8	3.9 ± 1.1	4.0 ± 0.9
Fasting Insulin * (pmol/L)	53 ± 37 ^a^	89 ± 53 ^b^	95 ± 48 ^b^	77 ± 49
Ghrelin * (pmol/L) Mean (95% CI)	171 (142–207) ^a^	111 (88–140) ^b^	112 (91–139) ^b^	132 (117–150)
Leptin (ng/mL) Mean (95% CI)	27 (20–38)	33 (20–54)	33 (23–47)	31 (25–38)
Adiponectin * (µg/mL) Mean (95% CI)	11 (9–13) ^a^	9 (7–11) ^a^	7 (6–8) ^b^	9 (8–10)
Plasma vitamin C * (µmol/L)	57 ± 14 ^a^	48 ± 16 ^b^	41 ± 18 ^b^	49 ± 17

Values represented as mean ± SD unless stated otherwise. *All *p* values from ANOVA tests. Groups sharing a common subscript letter denotes the study groups that do not differ significantly from each other at the 0.05 level based on characteristics from Post Hoc analysis. Log conversion was carried out for Ghrelin, Leptin, Adiponectin, and hs-CRP. Note: There was missing data from three participants for plasma vitamin C (2 × NGT and 1 × prediabetes).

**Table 3:** Dietary intake of participants classified as having normal glucose tolerance (NGT) (*n* = 35), prediabetes (*n* = 25), and T2DM (*n* = 29).

Total Daily Dietary intake	NGT	Prediabetes	T2DM	Total
Energy (KJ)	8192 ± 2336	8430 ± 2260	8033 ± 2416	8204 ± 2321
Fibre (g)	24 ± 9	25 ± 8	27 ± 9	25 ± 9
Protein (% of Energy)	17 ± 3	18 ± 4	17 ± 3	17 ± 3
Fat (% of Energy)	37 ± 6	39 ± 8	36 ± 7	37 ± 7
Carbohydrate (% of Energy)	44 ± 6	40 ± 8	44 ± 8	43 ± 7
Dietary Vitamin C Intake (mg)	103 ± 76	94 ± 58	101 ± 61	100 ± 66

Values represented as mean ± SD unless stated otherwise. Note: There was missing data from one participant for dietary information (1 × prediabetes). There were no significant differences between the study groups for any of the dietary intake measures.

**Table 4:** Pearson correlations of plasma vitamin C, glycaemic indices, hormones, lipids, high sensitivity C-reactive protein, and anthropometric measures.

Measure	Pearson Correlation
Fasting Glucose (mmol/L)	−0.411 ***
HbA1c (mmol/mol)	−0.334 ***
Total Cholesterol (mmol/L)	0.093
Triglycerides (mmol/L)	−0.322 **
Cholesterol (HDL)	0.295 **
Cholesterol (total/HDL)	−0.214 *
Cholesterol (LDL) calculated	0.086
Insulin (pmol/L)	−0.353 **
hs-CRP (mg/L)	−0.333 **
Ghrelin (pmol/L)	0.295 **
Leptin (ng/mL)	−0.183
Adiponectin (ng/mL)	0.202
BMI (kg/m2)	−0.446 ***
Waist-to-Hip Ratio	−0.274 *
Fat Mass (%)	−0.295 **

*** correlations significant at 0.001 level (2-tailed); ** correlations significant at the 0.01 level (2-tailed); * correlations significant at the 0.05 level (2-tailed).

**Table 5:** Multiple regression analysis showing significant associations with plasma vitamin C concentrations.

Measure	B	Lower 95% CI	Upper 95% CI	*p* Value
BMI	−0.9	−1.4	−0.4	0.001
Current Smoker	−21.9	−35.8	−7.9	0.003
Ex-Smoker	−4.9	−11.2	1.5	0.128
Fasting Glucose	−4.4	−7.1	−1.8	0.001
Dietary vitamin C	0.05	0.01	0.10	0.032

B: coefficient from the multiple linear regression model.
